# Determinants of malnutrition among pregnant and lactating women under humanitarian setting in Ethiopia

**DOI:** 10.1186/s40795-018-0222-2

**Published:** 2018-03-27

**Authors:** Betemariam Gebre, Sibhatu Biadgilign, Zinaw Taddese, Tsigereda Legesse, Mekitew Letebo

**Affiliations:** 1 International Medical Corps, Country Office, Khartoum, Sudan; 2 Public Health and Nutrition Consultant, P.O. Box 24414, Addis Ababa, Ethiopia

**Keywords:** Undernutrition, Pregnant and lactating women, Humanitarian, Ethiopia

## Abstract

**Background:**

Despite significant gains and progress in the last decade, malnutrition remains a major public health problem in Ethiopia. Pregnant and lactating women (PLW), along with children, are among the most vulnerable groups of population during emergencies and droughts. Identifying and targeting of PLW with malnutrition is among the priorities in humanitarian emergencies. However, there is dearth of evidence on PLW nutritional status and its determinants in humanitarian context.

**Methods:**

A community-based cross-sectional study was conducted in 10 kebeles of Rayitu district of Ethiopia in June 2013. A total of 900 PLW were assessed for malnutrition using mid-upper-arm circumference (MUAC).

**Result:**

Using MUAC < 21 cm as a criteria, 216 (24%) surveyed mothers were found to be malnourished. In multivariable logistic regression analysis, those mothers who did not received antenatal care (ANC) during their pregnancy had 1.83 higher odds of (adjusted odds ratio[AOR] = 1.83, 95% confidence interval [CI]:1.10,3.02) to be malnourished (MUAC < 21 cm) as compared to mothers who received ANC. Housewives had lower odds of (AOR = 0.59, 95 %CI: 0.37, 0.95) to be malnourished compared to those who engaged in as a pastoralist. Mothers belonging to families from which at least one person did not receive targeted supplementary feeding (TSF) in the 6 months before the study had lower odds of (AOR = 0.38, 95 %CI:0.23,0.62) to have acute malnutrition compared to those who lived in families who received TSF.

**Conclusions:**

Malnutrition is common among PLW in humanitarian settings, including those with ongoing interventions. Attending antenatal care, maternal occupational status and being a member of families who received TSF were factors associated with maternal nutritional status in this study. This signifies the need for sustainable solutions that address the high prevalence of malnutrition among PLW. Interventions targeting health system responses such as comprehensive nutrition education, support through antenatal care and women empowerment are recommended.

## Background

Despite significant gains and progress in the last decade, malnutrition remains a major public health problem in Ethiopia. Pregnant and lactating women (PLW), along with children, are among the most vulnerable groups of population during emergencies and droughts due to their higher nutritional needs and detrimental effects of poor nutrition on the health of the mothers and their children. Ethiopian Demographic Health Surveys (EDHS) 2000 and 2005 data revealed 30.5% and 26.9% of chronic energy deficiency among non-pregnant and non-postpartum women, respectively [1, 2]. Similarly, a prevalence of 27% from EDHS 2011 confirmed that the problem remained unchanged [3].

Malnutrition is known to increase the risk of poor pregnancy outcomes, including obstructed labor, premature or low-birth-weight (LBW) babies and postpartum hemorrhage. Severe anemia during pregnancy is associated with increased maternal mortality. Besides, malnutrition among mothers has an intergenerational effect, with repeating cycles of malnutrition and poverty in the long run [4].

Previous studies have established that malnourished pregnant women are at increased risk of having LBW infants [5, 6]. The link between LBW and poor health and nutritional outcomes later in life is also well established, with several studies reporting the association of LBW with malnutrition, poor growth and development, and increased morbidity and mortality in children [7–10]. Besides, poor nutrition during pregnancy, especially deficiencies of certain vitamins and minerals, have been associated with negative pregnancy outcomes for both the mother and the infant. Severe iron-deficiency anemia has been linked to preterm labor [11], poor anthropometric measures [12, 13] and birth asphyxia [13]. Studies on the impact of maternal malnutrition during lactation are rare. Several reports suggested a possible association of malnutrition among lactating mothers with production of smaller quantities of breast milk [14], and low levels of B vitamins, vitamin A and essential fatty acids in breast milk [7, 15, 16].

Maternal malnutrition is caused by complex interaction of a multitude of factors [17]. Severe illness, breastfeeding and having several children below 2 years of age are negatively associated with maternal nutritional status, while higher maternal age and socio-economic status, and household food security have positive effect. In addition, social factors, such as marital status, education, and income also have influence [18].

Screening and management of PLW with malnutrition have not always been thoroughly investigated, especially in the humanitarian context. There is a lack of consensus on which anthropometric measures to use, and the cut-off values to apply as criteria during screening and for admission of PLW to nutritional care programmes. The World Health Organization (WHO) is yet to provide a universally applicable indicator for nutritional assessment and supplementation of PLW in emergencies, although earlier recommendations suggested that all PLW could be eligible for targeted supplementary feeding (TSF). In humanitarian settings, mid-upper-arm circumference (MUAC) is often used for screening PLW, although cutoffs for defining acute malnutrition or maternal risk of having LBW infants varied. The broadly accepted SPHERE Guidelines also recommend the use of MUAC for PLW screening and entry of PLW into feeding programmes. According to these guidelines, the MUAC values associated with adverse outcomes vary from country to country, typically ranging from 21 cm to 23 cm and values < 21 cm could be used for identifying and targeting PLW at risk for growth retardation [19]. The Ethiopian Emergency Nutrition Coordination Unit (ENCU) suggests the same(< 21 cm) cutoff for admission of mothers to supplementary feeding programs in emergencies [20]. In addition to these dilemma around the screening of mothers in humanitarian context, there is a also paucity of published evidence reporting maternal nutritional status in humanitarian settings. The aim of this study is therefore to help bridge the evidence gap on the magnitude and determinants of maternal nutrition in humanitarian settings in general and Ethiopian context, in particular.

## Methods

### Study setting

The study was conducted in Rayitu Woreda (District), Bale Zone of Oromia Regional State in Ethiopia. Rayitu is bordered by Sewena and Ginir Districts on the North, Sewena District on the East, Goro to the West and Somali Regional State and Goro District on the South [21]. Currently, Bale zone is divided into 20 woredas. The zone comprises of several mountain ranges, massifs and plateaus, and is dominated by heavy precipitations. It has different climatic zones ranging from semi-arid to afro-alpine moorland, which makes the zone conducive for the existence of various flora and fauna within a relatively small area. Rayitu is one of the 20 woredas in Bale Zone of the Oromia Regional State that are classified as Bale Pastoral Livelihood Zone. Crop production is completely rain-fed. In normal years, the woreda receives annual rainfall in the range of 425–1300 mm. The area is marginal for agricultural production and suffers a food deficit every year. Households depend on both livestock and crop production. The main types of livestock are cattle, sheep, goats and camels. Seasonal livestock migration to the major rivers to seek pasture and water is common during the long dry season (Bona).

### Study participant, design and sampling

A community-based cross-sectional study was carried out using quantitative study design in June 2013. The target population in this study consists of pregnant women, lactating mothers and primary caregivers of children 6–23 months of age. A total of 1402 households were surveyed. Ten Kebeles (the smallest administrative units in government structure) were selected randomly from the list of Kebeles and then households were divided among Kebeles based on probability proportional to size (PPS). The allocation of individuals to Kebeles was also made based on PPS sampling methodology. Once the number of households in the Kebeles was determined, the households with the target population to be interviewed were picked from the household list of the Kebele administration. The actual location of the start of the survey in each area was decided based on random walk approach by spinning a pen to select the direction of the first household.

### Data collection procedures

Recruitment of the research team was carried out in collaboration with local health institutions. Experienced individuals who had conducted similar interviews were recruited. The principal investigators checked the activities of each team daily. Three principal investigators coordinated the overall data collection process. A total of 21 trained health professionals collected the data for 5 days.

### Measurements

Face-to-face interviews were held using a structured and field-tested questionnaire to collect data on socio-demographic and economic characteristics of the women and their households and exploring reproductive health issues. Nutritional status of PLW was determined through measurement of MUAC, which is commonly used approach in diagnosing acute malnutrition among PLW in humanitarian setting. MUAC of each woman was measured at the mid-point between the tips of the shoulder and elbow of the left arm using non-elastic, non-stretchable MUAC tapes. Measurements were recorded to the nearest 0.1 cm.

In this study, acute malnutrition or wasting, defined as MUAC < 21 cm, was the dependent variable. This cut-off point was used based on the SPHERE Guidelines [19] and national protocol [20], both of which recognize it as an appropriate level for identifying mothers who are at risk for giving birth to infants with growth retardation in humanitarian settings. Variables considered to build the regression model were age, educational status, marital status, and occupation of the mothers, annual income of the household, attending antenatal care (ANC), type of assistance received by members of the household in the preceding 3 months and receipt of TSF food rations or safety net food rations by any family members in the previous 6 months.

The English version of the designed questionnaire was translated into the local language of Oromiffa and checked to ensure equivalence between the English and Oromiffa versions. Pretest of the questionnaires was carried out before conducting the study. Training sessions provided to data collectors and supervisors focused on data collection procedures, quality, interviewing techniques and related issues. Apart from the two-day training and field practice, supervisors reviewed the collected data daily in order to identify errors, omissions and inconsistencies. Data was checked for completeness, accuracy and clarity by the study core team and supervisors. The consistency of data was assessed by double entering 10% of the responses at the end of data entry.

### Statistical analysis

Data entry, coding, cleaning and analysis were carried out using SPSS version 19 and STATA 12.0 (Stata Corporation, College Station, TX). Two experienced data entry clerks were involved in the process after receiving orientation on the survey questionnaires. Descriptive statistics were used to assess basic respondent characteristics. Variables explored in assessing determinants of maternal nutritional status were: socio-demographic characteristics (maternal age, educational status, marital status, ethnicity, religion and occupation and annual household income). Access to and utilization of health services (antenatal care and household visit by community health workers) and food assistance related variables (type of assistance mothers or other household members received, receipt of TSF or safety net food rations in the preceding 6 months) were also examined.

Variables with a *p*-value < 0.05 in the bivariate analysis and those variables which frequently showed significant association in previous studies, regardless of the *p*-value in the current study, were modeled into the multivariable logistic regression analysis to assess the determinant of maternal nutritional status. Both crude odds ratio (COR) and adjusted odds ratio (AOR) with corresponding 95% Confidence Interval were reported to show the nature of associations observed. In multivariable analysis, variables with a p-value of < 0.05 were considered as statistically significant.

## Result

### Participants

A total of 900 PLW were identified from 1402 households visited during the study. Detailed analysis of the socio-demographics characteristics of the respondents showed that close to one-third of them (30%) were in the 26–30-year age group. A quarter (25.6%) of the mothers were 20 years of age or younger. Regarding educational status, only 11.5% of the PLW reported that they were had formal schooling. Majority (94.2%) of the respondents were married. Most (99%) of the mothers were ethnic Oromo, and the same proportions were Muslims. Majority of the respondents were housewife (61%), pastoralist (32%) or farmers (7%). About 57% of the households had an average income which is less than the average family income which was 8770 Birr (See Table 1).

**Table 1:** Background characteristics of respondent in Rayitu District, 2013

Variables	Frequency (Percentage)		*P* value
	Malnourished (MUAC< 21 cm)	Normal (MUAC ≥21 cm)	
Age of Mothers/Caretakers			
15–20 yrs	40(18.78)	191(28.21)	0.105
21–25 yrs	71 (33.33)	179(26.44)	
26–30 yrs	67(31.46)	202(29.84)	
31–35 yrs	24(11.27)	68(10.04	
36–40 yrs	9(4.23)	33(4.87)	
41–45 yrs	2(0.94)	4(0.59)	
Mothers education status			
Illiterate	195(90.28)	601(87.87)	0.334
literate	21(9.72)	83(12.13)	
Mothers marital status			
Single	5(2.42)	23(3.44)	0.465
Married	202(97.58)	646(96.56)	
Mothers Occupation			
Pastoralist	93(44.08)	191(28.17)	< 0.001
Farmer	17(8.06)	41(6.05)	
Housewife	101(47.87)	446(65.78)	
Mothers ethnicity			
Oromo	213(99.07)	680(99.71)	0.222
Mothers Religion			
Muslim	214(100)	671(99.26)	0.207
Annual income of the household			
Below the mean(< 8770 Birr)^a^	132(71.74)	382(60.83)	0.007
Above the mean(≥8770 Birr)	52(28.26)	246(39.17)	
Family size			
≤ 2	1(0.47)	5(0.80)	0.841
3–5	117(54.93)	324(51.76)	
6–9	85(39.91)	267(42.65)	
≥ 10	10(4.69)	30(4.79)	

^a^Exchange rate I USD = 20.1 Ethiopian birr (ETB)

### Maternal nutritional status

**Fig. 1:**
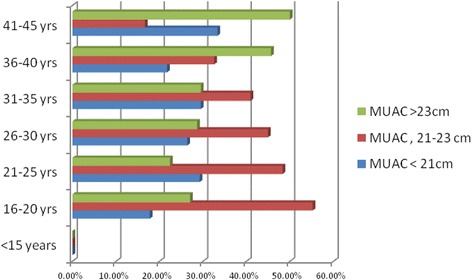
Maternal nutritional status (PLW) MUAC measurement by age of mothers in Rayitu District, 2013

Mid-upper arm circumference (MUAC) was used to assess nutritional status of PLW and determine the prevalence of malnutrition among mothers in the study. A total of 900 pregnant and lactating mothers were measured using MUAC tape. Accordingly, 216 (24%) of the mothers had a MUAC measurement of 20.9 cm or lower while 420 (46.67%) of them fall under the category of 21–23 cm. It is also noted that more than a quarter of the mothers 264 (29.33%) had a MUAC measurement of 23.1 cm or above. Overall, the mean MUAC measurement was 22.6 cm (with standard deviation of ±2.47 cm). Thus, based on the MUAC cut-off points provided in the current national guideline on malnutrition, 24% of the surveyed mothers require admission to feeding programs for malnutrition. Figure 1 shows maternal nutritional status with MUAC measurement by age.

### Determinants of maternal malnutrition

Bivariate and multivariable logistic regression analyses were carried out to determine factors associated with malnutrition among PLW in the study population. Findings are presented in Table 2. Accordingly, three variables were found to be associated with acute malnutrition as measured by MUAC (taking 20.9 cm as a criterion), in both the bivariate and multivariable analyses: attending antenatal care (ANC), benefiting from targeted supplementary feeding (TSF) in a form of food rations and occupational status of mothers. In multivariable logistic regression analysis, those mothers who did not received antenatal care (ANC) during their pregnancy were 1.83 times more likely (AOR = 1.83, 95%CI: 1.10, 3.02) to be malnourished (MUAC < 21 cm) as compared to mothers who received ANC. Housewives were 41% less likely (AOR = 0.59, 95 %CI: 0.37, 0.95) to be malnourished compared to those who engaged in as a pastoralist. Mothers belonging to families from which at least one person did not receive targeted supplementary feeding (TSF) in the 6 months before the study were 62% less likely (AOR = 0.38, 95 %CI: 0.23, 0.62) to have acute malnutrition compared to those who lived in families who received TSF.

**Table 2:** Bivariate and multivariable logistic regression analysis for assessing factor associated with maternal nutritional status in Rayitu District, June 2013

Variables	Frequency (Percentage)		Crude odd ratio	*P* value	Adjusted odd ratio^a^	*P*-value
	Malnourished (MUAC< 21 cm)	Normal (MUAC ≥21 cm)				
Attending antenatal care in the health facility						
Yes(ref)	139(66.19)	479(71.07)	1		1	
No	71(33.81)	195(28.93)	1.25(0.90, 1.75)	0.179	1.83(1.10, 3.02)	0.019
In the last 3 months, which type of assistance you or any member of your household Received?						
None(ref)	2(1.19)	9(1.76)	1		1	
Food only	155(92.26)	420(82.35)	1.66(0.35, 7.77)	0.519	2.64(0.31, 22.6)	0.374
Cash only	2(1.19)	50(9.80)	0.18(0.02, 1.45)	0.107	0.42(0.03, 5.46)	0.507
Food and cash alternatively	9(5.36)	31(6.08)	1.31(0.24, 7.17)	0.758	1.50(0.15, 14.9)	0.729
Did anyone in the family receive TSF food rations in the last 6 month?						
Yes(ref)	85(50.30)	149(28.93)	1		1	
No	84(49.70)	366(71.07)	0.40(0.28, 0.57)	< 0.001	0.38(0.23, 0.62)	< 0.001
Annual income of the household						
Below the mean(< 8770) (ref)*	132(71.74)	382(60.83)	1		1	
Above the mean(≥8770)	52(28.26)	246(39.17)	0.61(0.43, 0.87)	0.007	0.72(0.44, 1.19)	0.210
Did anyone in the family receive safety net food rations in the last 6 months?						
Yes(ref)	101(59.76)	354(68.74)	1		1	
No	68(40.24)	161(31.26)	1.48(1.03, 2.12)	0.032	1.06(0.66, 1.69)	0.815
In the last 6 months, were you visited by a field worker who talked to you about child and mother health?						
Yes(ref)	93(44.29)	353(52.37)	1		1	
No	117(55.71)	321(47.63)	1.38(1.01, 1.89)	0.041	1.09(0.69, 1.73)	0.716
Mothers Occupation						
Pastoralist(ref)	93(44.08)	191(28.17)	1		1	
Farmer	17(8.06)	41(6.05)	0.85(0.46, 1.58)	0.610	0.66(0.27, 1.59)	0.357
Housewife	101(47.87)	446(65.78)	0.46(0.33, 0.65)	< 0.001	0.59(0.37, 0.95)	0.032
Age of Mothers/Caretakers						
15–20 yrs(ref)	40(18.78)	191(28.21)	1		1	
21–25 yrs	71 (33.33)	179(26.44)	1.89(1.22, 2.93)	0.004	1.55(0.86, 2.78)	0.141
26–30 yrs	67(31.46)	202(29.84)	1.58(1.02, 2.46)	0.040	1.44(0.80, 2.58)	0.228
31–35 yrs	24(11.27)	68(10.04	1.68(0.95, 3.00)	0.076	1.99(0.94, 4.25)	0.072
36–40 yrs	9(4.23)	33(4.87)	1.30(0.58, 2.93)	0.524	2.49(0.86, 7.22)	0.094
41–45 yrs	2(0.94)	4(0.59)	2.39(0.42, 13.5)	0.325	–	

^a^The final model is adjusted for attending antenatal care in the health facility, type of assistance you or any member of the r household received, family receive TSF food rations in the last 6 month, annual income of the household, age of mothers/caretakers and anyone in the family receive safety net food rations in the last 6 months

## Discussion

This study is among the first to report on prevalence and determinants of malnutrition among PLW from a humanitarian setting in Ethiopia. In this study, a quarter of pregnant and lactating mothers were found to have acute malnutrition with the most stringent of criterion available (MUAC < 21 cm) and despite ongoing interventions in the area. Seasonal variation and the fact that this study was conducted during the known hunger season in the area might have contributed to the high rates of malnutrition among the PLW. Past studies from development settings in Ethiopia have reported a prevalence of 19.1% with a more lenient criterion of MUAC < 22 cm [22], clearly indicating that malnutrition rates could be higher during emergencies even after a period of interventions.

In this study, there seems to be some connection between receiving ANC and acute malnutrition among PLW. Similar findings were reported from development settings in the past, and the finding appears to be consistent with those. For example, in one study, frequency of attending ANC was a predictor of maternal nutritional status with those who had more visits having better nutritional indicators [23]. There are also studies which have directly linked frequency of antenatal care to having LBW deliveries [24] which is a reflection of maternal undernourishment. Relevant interventions should increase early use and effectiveness of antenatal care, increase food consumption during pregnancy and enhance behaviors that benefit fetal growth.

This study also revealed a higher prevalence of acute malnutrition among mothers whose family members received TSF. Given the fact that TSF programs target under five children and PLW with malnutrition during screenings in the selected TSF area, it is not surprising that families recently reached through this program had more mothers with malnutrition.

Another factor associated with acute malnutrition was occupational status of the mothers. Housewives were less likely to be malnourished as compared to those who mothers engaged in as a pastoralist. Past studies have shown similar results. In one study, the highest prevalence of underweight among mothers (44.3%) was found among women who were unskilled labourers and those who engaged in sales [25]. The prevalence of underweight among early childbearing mothers in Bangladesh is reported to be very high and is associated with occupational status of the mothers [26]. In another study, occupation of lactating mothers was strongly linked to their nutritional status [27]. However, there are also studies which reported the lack of association of maternal occupation to their nutritional status during pre- and post-harvest seasons [28].

Assistance in the form of food or cash transfer only, or food and cash transfer alternatively seems to play a huge role in preventing malnutrition among PLW in emergency context with families which received any type of support. In contrary to previous studies, in our study PLW from households with support of TSF were found to be more likely malnourished. This could be explained by the fact that, families with TFS support might be those who are highly food insecure. Unless the women in the current study are under direct TSF programme they cannot access the supplies. In reality TSF programmes are too strict that they monitor the supplies are only provided to those under the program. This signifies the importance of household based support than individual targets.

This study has some strengths and limitations. The strength is the identification and operation in emergency context where little similar research was done. The cross-sectional nature of the data used for this study makes it difficult to elucidate causality among variables and some variables may be missed like feeding practices, diversity, disease conditions, among others. This design also limited the time needed to collect some essential data such as market-related information at micro and macro level that could have provided a more comprehensive context to the analysis. Lack of generalizability is also anticipated as the study was carried out in a pocket geographical location of a country.

## Conclusions

Malnutrition rates among pregnant and lactating women remain high in emergencies context even with ongoing interventions. Receiving antenatal care, maternal occupational status and belonging to families which received TSF were factors associated with maternal nutritional status in this study. This signifies the need for sustainable solutions that address the high prevalence of malnutrition among PLW. Interventions targeting health system responses such as comprehensive nutritional education and support through antenatal care and women empowerment are recommended.

## Abbreviations

ANC: Antenatal care
AOR: Adjusted odds ratio
ENCU: Ethiopian emergency nutrition coordination unit
FANTA: Food and nutrition technical assistance
HEWs: Health extension workers
LBW: Low birth-weight
MUAC: Mid-upper-arm circumference
PLW: Pregnant and lactating women
PPS: Population-proportionate to size
SPSS: Statistical package for social sciences
WHO: World Health Organization
